# Effect of the G-308A polymorphism of the tumor necrosis factor (TNF)-α gene promoter site on plasma levels of TNF-α and C-reactive protein in smokers: a cross-sectional study

**DOI:** 10.1186/1471-2261-4-17

**Published:** 2004-10-14

**Authors:** Marie-Louise Gander, Joachim E Fischer, Friedrich E Maly, Roland von Känel

**Affiliations:** 1 Department of General Internal Medicine, University Hospital, Bern, Switzerland; 2 Institute for Behavioral Sciences, Federal Institute of Technology, Zürich, Switzerland; 3 Institute of Clinical Chemistry, University Hospital Zürich, Switzerland

## Abstract

**Background:**

Plasma levels of tumor necrosis factor (TNF)-α and of C-reactive protein (CRP) are elevated in smokers. Previous studies failed to show an association between the G-308A polymorphism in the promoter region of the TNF-α gene and coronary artery disease (CAD). We investigated whether smoking would interact with the TNF-α G-308A polymorphism in determining plasma levels of TNF-α and CRP.

**Methods:**

Study participants with a complete data set in terms of smoking and the TNF-α G-308A polymorphism were 300 middle-aged male and female industrial employees. After excluding 24 irregular smokers, analyses were performed on 198 "non-smokers" (life-long non-smokers or subjects who quit smoking >6 months ago) as compared to 78 "regular smokers" (subjects currently smoking >3 cigarettes/day). All subjects had a fasting morning blood draw to measure plasma levels of TNF-α and CRP by high-sensitive enzyme-linked immunosorbent assays.

**Results:**

The cardiovascular risk factor adjusted analysis regressing log-transformed CRP levels against smoking status, genotype, and smoking-status-genotype interaction revealed a significant main effect for smoking status (F_1,250 _= 5.67, p = .018) but not for genotype (F_1,250 _= 0.33, p = .57). The interaction-term between genotype and smoking status was not significant (F_1,250 _= 0.09, p = .76). The fully adjusted model with plasma TNF-α failed to show significant main effects for smoking and genotype, as well as for the smoking-status-genotype interaction.

**Conclusions:**

The findings suggest that the TNF-α G-308A polymorphism does not mediate the effect of smoking on plasma CRP levels. It remains to be seen whether other genetic polymorphisms along the inflammatory pathway may modulate vascular risk in smokers.

## Background

Coronary artery disease (CAD) is associated with chronic inflammatory processes in which blood-derived macrophages play a key role [1]. Tumor necrosis factor (TNF)-α is essentially produced by monocytes and macrophages, and, in turn, it is the strongest known paracrine activator of monocytes and macrophages [2]. Upon stimulation, these cells secrete a variety of products including interleukin (IL)-6 stimulating the liver to produce the acute phase reactant C-reactive protein (CRP) [3]. CRP is an acknowledged indicator of increased systemic inflammation across a wide range of diseases [4]. TNF-α and CRP both are found in considerable quantities in atherosclerotic lesions [5,6], and they have also been associated with increased cardiovascular risk in numerous large population-based studies [[7,8]; for review].

Smoking is one of the strongest cardiovascular risk factors for atherosclerotic diseases [9]. Several studies have revealed increased plasma levels of TNF-α and of CRP in smokers as compared to non-smokers [10,11], suggesting that part of the coronary risk associated with smoking may relate to increased inflammatory activity. However, the prevalence of cardiovascular disease varies substantially among smoking individuals [12]. This could indicate that genetic factors are important determinants of the biological pathways linking smoking with cardiovascular disease risk [13]. In fact, it has been recently shown that the CC polymorphism in the promoter region of the CD14 gene (CD14 -159C/T) was associated with common carotid artery intima-media thickness in smokers, but not in non-smokers [14]. Similarly, in young and healthy individuals carrying the C allele of the interleukin-6 promoter polymorphism -174 genotype, those who smoked had higher leukocytes, lymphocytes, and monocytes than those who did not smoke [15].

Another candidate polymorphism that might mediate the cardiovascular risk with smoking, and that is at the initiation of the inflammatory cascade, is the G-308A polymorphism of the TNF-α gene promoter site. The TNF-α G-308A polymorphism is a single base pair polymorphism located at position 308 in the TNF-α gene that maps to human chromosome 6 (p21.1-p21.3) resulting in substitution of the nucleotide adenine (A) for guanine (G) [16]. In the present study, we investigated the possible impact of this polymorphism on the association between smoking severity and plasma levels of TNF-α and CRP *in vivo*.

While the TNF-α G-308A polymorphism has been associated with increased production of TNF-α *in vitro *[17,18], previous studies failed to show an association of this polymorphism with CAD [19-25]. However, there is a dearth of data regarding possible interactions between environmental risk factors for cardiovascular disease (e.g., smoking) and the G-308A polymorphism on plasma levels of proinflammatory markers. It is conceivable that the TNF-α G-308A polymorphism alone is of negligible importance in CAD, but that the presence of certain environmental conditions (i.e., exposure to tobacco smoke) and specific alleles may influence CAD risk [26-28].

If alleles are randomly distributed between smokers and non-smokers, case-control studies not explicitly investigating the possible environmental-genetic interaction might fail to unravel the role of genetic polymorphisms in CAD. Therefore, we speculated that smoking would affect plasma TNF-α and CRP levels depending on the TNF-α genotype. If so, the genotype might affect the rate of disease progression rather than the existence of atherosclerotic lesions.

## Methods

### Study participants

The study was conducted as part of a larger project in an airplane manufacturing plant in southern Germany. From a total of 1,760 employees, participation was offered to a representative sample of 647 men and women. Of those, 537 (accrual rate 83%) volunteered to participate. The Institutional Review Board approved the study protocol. All 537 subjects completed questionnaires on medical and psychosocial health status. A subsample of 332 subjects agreed to have a variety of biological measures assessed. Three hundred of these individuals had complete data on smoking status, smoking history and the TNF-α G-308A gene polymorphism.

Based on self-reported smoking history, we categorized life-long non-smokers or those, who had quit smoking for at least 6 months as „non-smokers" (n = 198), smokers who were currently smoking >3 cigarettes per day as "regular smokers" (n = 78), and smokers reporting to consume up to 3 cigarettes/day or who had stopped smoking for less than 6 months as „irregular smokers" (n = 24). Because of the resulting small numbers of irregular smokers, the latter 24 individuals were excluded from further analyses. Of the remaining 276 individuals, we excluded those with a history suggestive of symptomatic atherosclerotic disease, individuals reporting intake of drugs or conditions that might affect CRP levels (including chronic inflammatory diseases such as active rheumatoid arthritis), and subjects for whom CRP data were missing because of occasional assay problems. Subjects reporting a positive history of elevated blood glucose were not excluded. This selection procedure resulted in a final dataset of 261 subjects.

### Experimental protocol

Data were collected on two occasions: First, subjects completed a medical questionnaire and examination. The medical assessment consisted of a 96-item questionnaire assessing the medical history and smoking behavior. The questionnaire was based on the Nurses Health Study [29] with questions asking for smoking behavior adapted from the MONICA study [30].

After completion of questionnaires, subjects had a 15-min rest period while sitting. Thereafter, systolic and diastolic blood pressure (BP) were measured twice within 5 min by sphygmomanometry, and the average of the two readings was computed. The waist-to-hip ratio was calculated based on waist circumference (as measured at its narrowest point between the ribs and iliac crest) and hip circumference (as measured at the maximal buttocks). Blood samples after overnight fasting were collected within one day to two weeks after having obtained medical data. Blood sampling was scheduled two hours after awakening to minimize circadian variation in variables of interest.

### Biochemical analyses

Venous blood was obtained using cooled (4°C) citrate tubes for the TNF-α assay. Plasma was snap-frozen after centrifugation until further processing. We chose high-sensitive enzyme-linked immunosorbent assays (ELISA) to measure plasma concentrations of TNF-α (Quantikine HS, R&D Systems Europe, Abington, United Kingdom) and of CRP (detection limit 0.1 mg/l; Immunolite, DPC Biermann GmbH, Germany). In contrast to standard CRP assays, the high-sensitivity assay for CRP allows stratification of subjects with CRP levels below the range used for infectious disease workup [31]. Moreover, it has been argued that the high-sensitive CRP assay is required for risk assessment of cardiovascular disease [32]. Low-density lipoprotein cholesterol (LDL-C) cholesterol and high-density lipoprotein cholesterol (HDL-C) as well as hemoglobin A1c (HbA1c) were determined by a commercial laboratory (Synlab, Augsburg, Germany) applying standard procedures.

### Gene analysis

To determine the TNF-α -308 G/A gene polymorphism, we extracted genomic DNA from the leukocyte-containing pellets remaining after centrifugation of coagulated blood using the QIAmp DNA Blood Mini Kit (Qiagen, Hilden, Germany). The TNF-α G-308A polymorphism was assessed by fluorescent real-time polymerase chain reaction with melting curve analysis on a LightCycler (Roche Diagnostics, Rotkreuz, Switzerland) using the TNF-α G-308A ToolSet for LightCycler (Genes-4U, Neftenbach, Switzerland) containing specific primers and fluorescent mutation detection oligonucleotide probes, in conjunction with the Roche Light Cycler Hyb Probe Master Mix (Roche Diagnostics, Rotkreuz, Switzerland) according to the manufacturer's protocols. For statistical analyses, we used the following groups: a) the GG variant, and b) the rarer AA and GA genotypes combined.

### Statistical analyses

Descriptive data are presented as means ± SD or as median and interquartile range for severely skewed data. To approximate a normal distribution, we log transformed TNF-α and CRP values. General linear models were employed to elucidate the proportion of variance explained of log-transformed plasma TNF-α and CRP values (dependent variables). Independent variables were smoking status (regular smokers vs. non-smokers), gene variant, and an interaction term between smoking status and gene variant. Following this crude analysis, we entered possible covariates (age, gender, self-reported physical exercise, self-reported alcohol intake, gender, BP, and lipoproteins) into the equation. Results were considered statistically significant at the p ≤ .05 level; all tests were 2-tailed. To minimize possible type II errors when assessing interaction terms, we considered interaction terms when the F-statistic on the interaction term had a p-value < 0.2 [33]. All regression and variance analyses were performed using generalized linear models (PROC MIXED) to account for the unbalanced nature of the data (SAS version 8.2, SAS Inc, Cary, NC). Analyses were repeated including the small group (n = 24) of irregular smokers either with the non-smokers or with the regular smokers. None of these additional analyses changed our main results to a relevant degree. Therefore, only the results comparing non-smokers with regular smokers are reported.

## Results

### Study population and smoking status

Table 1 compares health variables between non-smokers (n = 198), irregular smokers (n = 24) and regular smokers (n = 78). Regular smokers tended to be younger (p = 0.09), and they had significantly lower HDL-C levels (p = 0.01) than non-smokers. Regular smokers also had higher plasma levels of CRP than non-smokers (median 1.75 mg/l vs. 1.0 mg/l, p = 0.006). In our study sample, irregular smokers had plasma levels of CRP comparable to non-smokers (median 0.81 vs. 1.0 mg/l, p = 0.94). TNF-α was not significantly different between regular smokers and non-smokers.

**Table 1:** Subject characteristics in relation to smoking status

	**Smoking status**			
**Variable**	**Non-smokers for >6 months **n = 198	**Irregular smokers **n = 24	**Regular smokers **n = 78	P-value^a^

Gender [% male]	86	80	88	0.52
Age [years]	42.1 ± 9.0	40.6 ± 9.2	40.0 ± 9.6	0.09
LDL [mg/dl]	122.3 ± 28.5	117.4 ± 30.4	115.9 ± 28.3	0.10
HDL [mg/dl]	45.3 ± 10.8	47.1 ± 8.3	41.4 ± 9.2	0.01
Systolic blood pressure [mmHg]	132.9 ± 15.2	128.7 ± 9.9	131.2 ± 13.6	0.43
Diastolic blood pressure [mmHg]	82.9 ± 9.7	79.2 ± 8.0	81.6 ± 8.3	0.33
Glycosylated hemoglobin [%]	5.15 ± 0.5	5.1 ± 0.4	5.25 ± 0.4	0.12
Tumor necrosis factor-α [ng/l]	1.8 ± 0.5	1.8 ± 1.0	2.2 ± 2.5	0.61
C-reactive protein [mg/l]	1.0 (0.47–1.9)	0.80 (0.5–2.0)	1.75 (0.7–3.0)	0.006

^a ^P-values for comparisons between non-smokers and regular smokers. Tumor necrosis factor-α and C-reactive protein (CRP) levels were compared using the Wilcoxon test; for all other continuous variables the Student *t*-test was employed. Values are means ± SD except for CRP where the median and interquartile range is provided. LDL = Low-density lipoprotein cholesterol, HDL = High-density lipoprotein cholesterol

### Gene distribution

After excluding irregular smokers, we found the GG wild type polymorphism in 203 subjects (74%); GA heterozygote were 70 participants (25%) and AA homozygote were 3 participants (1%). Calculated allele frequencies amounted to 0.86 for the G allele and to 0.14 for the A allele. Regular smokers were slightly though not significantly more frequent amongst subjects with the GG genotype than amongst individuals carrying the GA or AA genotype (odds ratio 1.41; 95% CI 0.77–2.6, p = 0.26). Table 2 compares individuals with the GG wild type with participants having the GA or the AA genotype. The table reveals that genotype was not associated with any of the examined health variables in crude bivariate comparisons, including plasma levels of TNF-α and CRP.

**Table 2:** Subject characteristics in relation to the TNF-α G-308 polymorphism

	**GG **n = 203	**GA/AA **n = 73	**P-value**
Gender [% male]	87	86	0.73
Age [years]	41.0 ± 9.1	42.9 ± 9.4	0.37
Regular smokers [%]	28.3	21.8	0.26
Low-density lipoprotein cholesterol [mg/dl]	119.5 ± 32.6	124.8 ± 29.1	0.22
High-density lipoprotein cholesterol [mg/dl]	44.1 ± 11.9	44.62 ± 10.9	0.76
Systolic blood pressure [mmHg]	131.7 ± 11.7	134.6 ± 17.4	0.18
Diastolic blood pressure [mmHg]	82.5 ± 11.1	82.7 ± 8.1	0.87
Glycosylated hemoglobin A1c [%]	5.1 ± 0.5	5.2 ± 0.6	0.18
Tumor necrosis factor-α [ng/l]	1.7 (1.3–2.2)	1.9 (1.4–2.2)	0.39
C-reactive protein [mg/l]	1.04 (0.53–2.36)	1.05 (0.49–3.14)	0.35

Tumor necrosis factor (TNF)-α and C-reactive protein (CRP) levels were compared by the Wilcoxon test; for all other continuous variables the Student *t*-test was employed. Values are means ± SD except for TNF-α and CRP where the median and interquartile range is provided.

### Genotype, smoking status, TNF-α and CRP levels

The adjusted analysis regressing log-transformed CRP levels against smoking status, genotype, and smoking-status-genotype interaction revealed a main effect for smoking status (F_1,250 _= 5.67, p = .018), but not for genotype (F_1,250 _= 0.33, p = .57). The interaction-term between genotype and smoking status failed to gain significance (F_1,250 _= 0.09, p = .76), indicating that the effect of smoking on plasma levels of CRP is not affected by the TNF-α -308G/A polymorphism (Figure). Of the considered covariates, gender (F_1,250 _= 12.5, p = .0005), age (F_1,250 _= 4.55, p = .03), and HDL-C (F_1,250 _= 3.4, p = .065) were associated with CRP levels, but not BP or LDL-C. Men, older individuals, and those with lower HDL-C had higher plasma levels of CRP.

**Figure 1:**
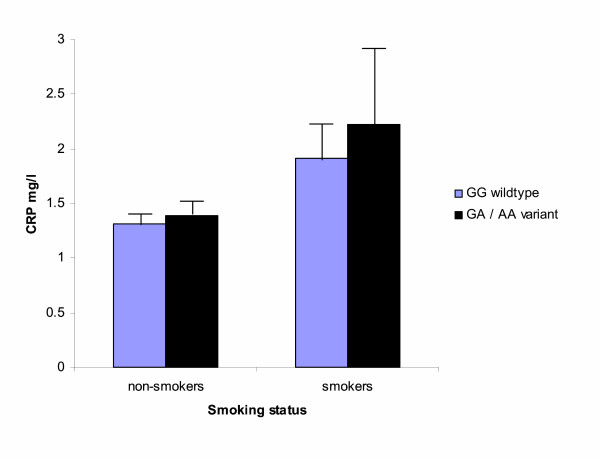
The figure depicts plasma levels of C-reactive protein (CRP) in relation to smoking status and the TNF-α G-308 polymorphism in the final sample of 261 subjects. CRP levels are anti-log transformed least square means estimates from the fully adjusted model controlling for age, gender, blood pressure and blood lipids. Error bars denote the standard error of the mean estimate. Simple effects comparing genotypes across smokers were not significant (p = .14).

The fully adjusted model with plasma levels of TNF-α also failed to show significant main effects for genotype (F_1,246 _= 1.08, p = .30) and for smoking (F_1,246 _= 0.33, p = .56), as well as for the smoking-status-genotype interaction. However, the adjusted model revealed a significant main effect for HDL-C (F_1,246 _= 20.7, p < .0001), suggesting that individuals with higher plasma levels of TNF-α had lower HDL-C. Post-hoc analyses revealed no interaction between smoking status and HDL-C or genotype and HDL-C.

## Discussion

Genes, health-behavior and the psychosocial environment interact to determine whether or not, and, if at all, how rapidly silent atherosclerosis will progress to the clinical manifestation of CAD [34,35]. The development of coronary artery sclerosis is a life-long process that probably has its onset in childhood [36]. Particularly, as suggested by post mortem studies, inflammation-related endothelial damage plays an important role in atherosclerosis onset and progression early in life [37]. The understanding of atherosclerosis as an inflammatory disease [1] has kindled much interest in a number of genetic polymorphisms coding for inflammatory molecules potentially related to CAD [38].

While plasma levels of the proinflammatory cytokine TNF-α are regulated by several polymorphisms of the TNF-α gene [39], it is the TNF-α G-308A gene polymorphism which has been most intensely scrutinized as one candidate polymorphism underlying CAD [8]. Plasma TNF-α levels predicted second myocardial infarction [40], and have been associated with common carotid intima-media thickness [41]. TNF-α also stimulates the liver to produce CRP [8], which, itself, has been shown to predict coronary risk in numerous population based studies [7].

Interestingly, blood cells from individuals who carry the A allele of the TNF-α G-308A gene polymorphism express more TNF-α in *vitro *upon stimulation with lipopolysaccharide than cells from individuals being homozygous for the G allele [18]. Despite this association, several studies did not find a significant association between the TNF-α G-308A gene polymorphism and incident CAD [19-25]. There are, however, no studies examining whether established cardiovascular risk factors might interact with the TNF-α G-308A gene polymorphism in determining plasma levels of TNF-α and eventually CRP downstream in the inflammatory cascade. We thus investigated the effect of an interaction between smoking severity and the G-308A polymorphism of the TNF-α gene on plasma levels of these two proinflammatory markers. Our specific hypothesis was that there was a cumulative increase of TNF-α and CRP related to the TNF-α G-308A polymorphism in subjects who regularly smoke as compared to non-smokers.

In spite of two recent studies, which found an interaction between smoking and polymorphisms of molecules participating in the inflammatory response [14,15], the results from the present study fail to support our hypothesis. More precisely, we found that the interaction between smoking status and the TNF-α G-308A polymorphism did not significantly affect plasma levels of TNF-α and CRP in both unadjusted and adjusted analyses. Also, there was no main effect for the polymorphism investigated in terms of plasma levels of TNF-α and CRP. On the other hand, although not an aim of our study, we confirmed previous findings of increased plasma CRP in regular smokers as compared to non-smokers [11], while, rather unexpectedly, plasma levels of TNF-α were not different between smokers and non-smokers.

It must be noted that our findings are preliminary, and, they do not allow us to reject the overall hypothesis of a smoking-gene interaction modifying inflammatory processes contributing to atherosclerosis initiation and progression [14,15]. For instance, because the number of homozygous carriers of the A allele in our study population was low reflecting low frequency of the AA genotype in the general population, we were unable to analyze whether there might be a "dose-response" relationship between the A allele dosage and CRP levels. Larger sample sizes are clearly needed to detect a potential difference in regulation of proinflammatory markers in plasma across the GG, AG, and AA polymorphism and with respect to their interaction with different cardiovascular risk factors. This reasoning becomes even more obvious with respect to the higher absolute difference in the mean estimates of plasma CRP levels between the GG and the GA/AA genotypes in smokers as compared to non-smokers (Figure). A highly powered study might raise the odds of this absolute difference to become statistically significant. Moreover, the biological plausibility of our hypothesis was straightforward given the important role of smoking, inflammation and their link in CAD [9-11]. However, we do not know in how far interactions between smoking, the TNF-α G-308A polymorphism, and other polymorphisms of molecules involved in the inflammatory pathways not investigated in our study [14,15] might affect plasma TNF-α and CRP levels in an unexpected way.

The lack of a difference in plasma TNF-α levels between subjects with the A allele as compared to those homozygous for the G allele stands in contrast to previous *in vitro *studies [17,18]. However, aside from a power issue, our measured values of TNF-α levels only slightly exceeded the assay's sensitivity limit, incurring a larger chance of measurement error. We may speculate that a relation between gene variant, smoking, and circulating TNF-α levels might have been uncovered among patients with atherothrombotic disorders or other inflammatory conditions. Moreover, we measured systemic TNF-α; circulating TNF-α may not necessarily reflect TNF-α secretion at sites of confined subendothelial atherosclerotic lesions, where regulatory polymorphisms are most likely to affect reactions of immune cells. Finally, interactions also involving IL-6 polymorphisms [15] may play a role in the association between smoking, elevated CRP, and increased CAD risk. Future studies thus may want to investigate whether IL-6 polymorphisms might be associated with plasma CRP levels and whether they interact with TNF-α and CRP in smokers.

## Conclusions

Our study suggests that both plasma TNF-α and CRP levels are not regulated by an interaction between smoking and the G-308A polymorphisms of the TNF-α gene promoter site. We thus remain far from adopting a clinical practice that would counsel smokers to quit smoking based on a particular gene polymorphism. Nonetheless, our results do not refute the overall hypothesis that genetic polymorphisms along the inflammatory pathway may account for the differential effect of tobacco consumption on the cardiovascular risk in individuals who smoke.

## Competing interests

The authors declare that they have no competing interests.

## Authors' contributions

MG participated in the design of the study and drafted the first version of the manuscript.

JF participated in the design of the study, in data acquiring, performed the statistical analyses, and critically revised the manuscript.

FM carried out and supervised the molecular genetic studies.

RvK participated in the design of the study and wrote the final version of the manuscript.

All authors read and approved the final manuscript.

## Pre-publication history

The pre-publication history for this paper can be accessed here:


